# Associations of S-Adenosylmethionine and S-Adenosylhomocysteine with Hepatocellular Carcinoma

**DOI:** 10.3390/metabo15110740

**Published:** 2025-11-13

**Authors:** Naana N. Yalley, Sebastian M. Armasu, Winnie Z. Fan, Irene K. Yan, Fowsiyo Y. Ahmed, Per Stål, Lewis R. Roberts, Tushar Patel, Samuel O. Antwi

**Affiliations:** 1 Division of Epidemiology, Department of Quantitative Health Sciences, Mayo Clinic, Jacksonville, FL 32224, USA; yalley.naana@mayo.edu; 2 Division of Clinical Trials and Biostatistics, Department of Quantitative Health Sciences, Mayo Clinic, Rochester, MN 55902, USA; armasu.sebastian@mayo.edu (S.M.A.);; 3 Department of Cancer Biology, Mayo Clinic, Jacksonville, FL 32224, USA; yan.irene@mayo.edu (I.K.Y.); patel.tushar@mayo.edu (T.P.); 4 Division of Gastroenterology and Hepatology, Department of Internal Medicine, Mayo Clinic, Rochester, MN 55902, USA; ahmed.fowsiyo@mayo.edu (F.Y.A.); roberts.lewis@mayo.edu (L.R.R.); 5 Department of Upper GI Diseases, Karolinska University Hospital, SE-171 77 Stockholm, Sweden; per.stal@ki.se; 6 Department of Transplantation, Mayo Clinic, Jacksonville, FL 32224, USA; 7 Division of Gastroenterology and Hepatology, Department of Internal Medicine, Mayo Clinic, Jacksonville, FL 32224, USA

**Keywords:** hepatocellular carcinoma, HCC, s-adenosylmethionine, SAM, s-adenosylhomocysteine, SAH, metabolic dysfunction-associated steatotic liver disease, MASLD

## Abstract

**Background:** Hepatocellular carcinoma (HCC) is a leading cause of cancer-related deaths worldwide, increasingly arising in patients with metabolic dysfunction-associated steatotic liver disease (MASLD). Epigenetic dysregulation, particularly DNA methylation, has been implicated in MASLD-HCC development, yet the roles that the principal DNA methylation precursor metabolites, S-adenosylmethionine (SAM) and S-adenosylhomocysteine (SAH), play in this association are unclear. **Objective:** We investigated associations of circulating SAM, SAH, the SAM/SAH ratio, with MASLD-HCC. **Methods:** In a multi-center pilot case–control study, we evaluated 69 MASLD-HCC cases and 136 cancer-free MASLD controls. Plasma SAM and SAH levels were quantified by liquid chromatography–tandem mass spectrometry. Metabolite levels were categorized as greater than or less than the median based on distribution in controls. Logistic regression was used to calculate odds ratios (ORs) and 95% confidence intervals (CIs), adjusting for age, sex, body mass index, smoking status, and type 2 diabetes. **Results:** MASLD-HCC cases had significantly higher plasma SAM levels (mean 121 vs. 96 nmol/L; *p* = 0.001) and SAM/SAH ratios (2.09 vs. 1.48; *p* = 6.42 × 10^−7^) than MASLD controls. In multivariable-adjusted models, elevated SAM levels (OR_≥median vs. <median_ = 2.76; 95% CI: 1.38–5.72) and higher SAM/SAH ratio (OR_≥median vs. <median_ = 2.30; 95% CI: 1.15–4.73) were associated with higher odds of MASLD-HCC. SAH alone was associated with MASLD-HCC. **Conclusions:** Higher plasma SAM levels and SAM/SAH ratios are independently linked to MASLD-HCC development. These metabolites might serve as noninvasive markers for HCC risk stratification in patients with MASLD and improve early detection efforts for MASLD-HCC.

## 1. Introduction

Hepatocellular carcinoma (HCC) accounts for about 70% of all primary liver cancers [1] and is the third leading cause of cancer deaths worldwide [2]. The major risk factors of HCC include hepatitis B virus (HBV) and hepatitis C virus (HCV) infections, aflatoxin B1 exposure, excessive alcohol intake, metabolic dysfunction-associated steatotic liver disease (MASLD) and its more severe form, metabolic dysfunction-associated steatohepatitis (MASH), cigarette smoking, obesity, and type 2 diabetes [3]. Despite significant advancements made in the diagnosis and treatment of HCC, it remains a highly fatal cancer with an overall 5-year survival rate of 22% [1]. The poor prognosis of HCC is due mainly to most patients being diagnosed at later stages of the cancer, coupled with limited treatment options at advanced tumor stages [1]. Enhancing both prevention strategies and early detection of HCC are important goals for improving patient outcomes.

S-adenosylmethionine (SAM) and S-adenosylhomocysteine (SAH) are essential precursor molecules involved in DNA methylation, a molecular mechanism implicated in HCC development [4,5,6]. SAM is the universal methyl donor used for DNA methylation [7] and is involved in about 85% of all methylation reactions [8]. SAM is formed primarily in the liver and is derived mainly from the amino acid methionine [9,10]. Methionine is often biosynthesized through metabolism of one-carbon nutrients, such as betaine (a choline metabolite) and 5-tetrahydrofolate (the primary circulating form of folate) [9,10]. The metabolic cycles of betaine, methionine, and 5-tetrahydrafolate intersect at a juncture where the amino acid homocysteine is converted to methionine, and 5-tetrahydrofolate and betaine are also converted to methionine [10,11]. Methionine is subsequently converted to SAM, which donates methyl (CH_3_) groups for methylation of the 5-carbon position of cytosine in DNA (i.e., DNA methylation) [12,13,14]. Following the transfer of CH_3_ from SAM for methylation, SAM is then converted to SAH, which is a potent inhibitor of cellular methylation reactions [12,13,15]. Thus, while SAM promotes methylation, SAH inhibits methylation reactions, and the SAM/SAH ratio reflects cellular methylation potential [15]. Because SAM and SAH are both biosynthesized primarily in the liver, the SAM/SAH ratio is often referred to as “hepatic methylation potential” [16,17] or “hepatic methylation index” [18,19]. However, there appears to be a delicate balance between SAM and SAH needed for normal DNA methylation reactions.

Although SAM serves as the universal methyl donor used for DNA methylation, it is important to recognize that DNA methylation accounts for only a small fraction of total hepatic SAM utilization [20,21]. In the liver, SAM participates in numerous essential metabolic reactions, including the synthesis of phosphatidylcholine via the phosphatidylethanolamine methyltransferase (PEMT) pathway, as well as the biosynthesis of creatine, carnitine, glutathione, and polyamines (spermine and spermidine) [18,21,22,23]. Also, SAM and SAH play key roles in liver regeneration, differentiation, and modulation of hepatic sensitivity to injury [22,24,25]. Therefore, alterations in plasma SAM and SAH levels may reflect broader disturbances in hepatic one-carbon metabolism or methyl group turnover, rather than changes limited to DNA methylation processes alone.

Low SAM and high SAH levels have been associated with DNA hypomethylation, which is linked to cellular oxidative stress, and protein misfolding resulting in endoplasmic reticulum (ER) stress and inflammation [17,18]. High SAH levels have also been linked to liver injury owing to the buildup of homocysteine in the liver [19]. Further, animal studies show that administration of SAM to carcinogen-treated rats inhibits hepatic tumorigenesis, whereas induction of chronically low SAM levels in rats induces hepatic steatosis and HCC development [19]. This suggests that low SAM levels may be involved in MASLD-related HCC development, at least in murine models. Even though plasma SAM levels have been shown to predict advanced liver fibrosis and poor Child–Pugh status in HBV-infected patients [26] and the SAM/SAH ratio has been proposed as a potential biomarker of liver disease severity [19], it remains unclear whether an altered SAM/SAH ratio influences susceptibility to HCC in humans, especially in the setting of metabolic abnormalities.

Here, we aimed to investigate whether plasma SAM or SAH level, or the SAM/SAH ratio, is associated with HCC development in patients with MASLD. We performed a pilot multicenter case–control study among patients with MASLD-HCC and cancer-free MASLD control patients, providing initial insights into potential roles of SAM and SAH in HCC development.

## 2. Methods

### 2.1. Data Source and Study Population

Details of the design and methods used for participant recruitment and sample collection have been published previously [4,27]. In brief, we obtained data and biospecimen from the following international sites: (1) the Karolinska University Hospital, Sweden; (2) the Barcelona Clinic Liver Cancer Group (BCLC), Hospital Clinic Barcelona and IDIBAPs, Barcelona, Spain; (3) Instituto de Investigación Sanitaria Biogipuzkoa (IISB), Donostia University Hospital, San Sebastian, Spain; and (4) the Mayo Clinic sites in Rochester, Minnesota and Jacksonville, Florida. The participating sites provided epidemiological data and blood samples on 673 MASLD-HCC cases and 763 cancer-free MASLD controls. However, plasma samples were only available for 70 MASLD-HCC cases and 216 MASLD controls. Prior to providing the data and biospecimens to Mayo Clinic, all sites were asked to exclude any patients with history of viral hepatitis (HBV, HCV), alcoholic liver disease or excessive alcohol intake (≥20 g/day), or have competing liver diseases (e.g., autoimmune hepatitis, Wilson’s disease, hemochromatosis, Budd–Chiari syndrome, and alpha-1 antitrypsin deficiency) leaving only patients with MASLD-HCC or MASLD controls [4,27]. The study sites also provided data on age at HCC diagnosis for cases or age at recruitment for controls, sex, race/ethnicity, type 2 diabetes, cigarette smoking history, body mass index (BMI), and amount of moderate alcohol consumption (<20 g/day). From the existing pool of 70 MASLD-HCC cases and 216 MASLD controls with plasma samples available, we performed a one-to-two frequency matching based on age, sex, and race, resulting in 70 MASLD-HCC cases and 140 MASLD controls being used for the SAM and SAH assay.

All participating sites previously received ethical approval from their local Institutional Review Boards (IRBs) that allowed samples collection and shipment, and all study participants provided informed written consent. For the present study, additional IRB approval was obtained from the Mayo Clinic IRB (IRB # 23-000005).

### 2.2. Mass Spectrometry Assay for SAM and SAH

Plasma samples were collected and stored at −80 °C until assayed by liquid chromatography coupled with electrospray ionization tandem mass spectrometry (HPLC-ESI-MS/MS), following the method described by Arning et al. [28]. In brief, 20 μL of plasma per participant was analyzed using HPLC-ESI-MS/MS. Each analytical run included calibration standards for SAM and SAH, along with isotopically labeled internal standards (^2^H_3_-SAM and ^2^H_4_-SAH) for quantitation. Sample preparation involved mixing 20 μL of plasma with 180 μL of internal standard solution (prepared in mobile phase A), followed by ultracentrifugation through a 10 kDa molecular weight cut-off filter. A 3 μL aliquot of the filtrate was injected into a Shimadzu Nexera LC system coupled to a Sciex 5500 QTRAP^®^ mass spectrometer (SCIEX, Framingham, MA, USA). Chromatographic separation was achieved on a 250 mm × 2.0 mm EZ-faast column (Phenomenex, Torrance, CA, USA) at a flow rate of 0.20 mL/min, using a binary gradient with a 10 min total run time. The mass spectrometer operated in positive ion mode with an ion spray voltage of +5000 V. SAM and SAH eluted at approximately 5.8 and 5.5 min, respectively, and were distinguished by their characteristic MS/MS fragmentation patterns. Calibration curves were linear over the range of 12.5–5000 nmol/L for both analytes [28].

A total of 70 MASLD-HCC cases and 140 MASLD controls were assayed, along with 10% duplicate samples (n = 19; 6 cases and 13 controls; 1 case and 1 control were triplicates). Concordance between duplicate measurements for SAM and SAH were 0.88 and 0.89, respectively. For duplicates, the mean value of the two measurements was used in downstream analyses. Samples identified as outliers (case n = 1, controls n = 4) were excluded based on quality control assessments, which included Bland–Altman plots, Rosner’s test for outliers, and visual inspection of metabolite distributions. This resulted in a final analysis set of 69 cases and 136 controls.

### 2.3. Statistical Analysis

Differences in participant characteristics were compared between cases and controls using the Wilcoxon rank sum test for continuous variables and chi-square tests for categorical variables. Associations of SAM, SAH, and the SAM/SAH ratio were assessed using both continuous variables and categorical variables. For the categorical variables, we used the median value of each exposure variable determined among controls as a cut point to dichotomize each variable into less than the median (reference category) versus greater than or equal to the median. The association of each exposure variable with MASLD-HCC risk was assessed using unconditional logistic regression to calculate odds ratios (ORs) and 95% confidence intervals (CIs) in univariable and multivariable models. In the multivariable model, we adjusted for age (continuous), sex, BMI (continuous), smoking history (never, former, current), and type 2 diabetes (yes, no). All statistical tests were two-sided, and a *p*-value < 0.05 was considered statistically significant. Statistical analyses were performed using the R software (version 4.4.1, R Foundation for Statistical Computing, Vienna, Austria).

## 3. Results

We analyzed a total of 205 samples, comprising 69 MASLD-HCC cases and 136 MASLD controls. Demographic and clinical characteristics of the participants are shown in Table 1. Briefly, no significant differences were observed between the cases and controls by age, sex, and type 2 diabetes status. However, the cases included a greater proportion of current or former smokers than controls. Mean SAM levels were higher in cases than controls (121 vs. 96 nmol/L, *p* = 0.001), but SAH levels did not differ significantly between the cases and controls (mean: 63 vs. 65 nmol/L, *p* = 0.11). The SAM/SAH ratio was higher in the cases than controls (mean: 2.09 vs. 1.48, *p* = 6.42 × 10^−7^).

**Figure 1:**
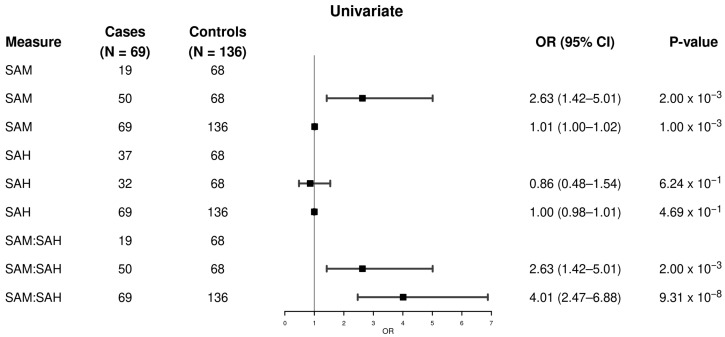
Univariate association of plasma SAM and SAH levels, and the SAM/SAH ratio with MASLD-HCC. The analysis included 205 samples (69 MASLD-HCC cases and 136 MASLD controls). Logistic regression models were used to calculate odds ratios, 95% confidence intervals, and *p*-values. Metabolite levels were analyzed both as continuous variables and as binary variables, using the median value among controls as the cutoff point (individuals with values below the median served as the reference group). Higher plasma SAM levels and SAM/SAH ratios were associated with increased odds of MASLD-HCC, whereas SAH levels showed no significant association with MASLD-HCC. Abbreviations: CI: confidence interval; OR: odds ratio; SAM: S-adenosylmethionine; SAH: S-adenosylhomocysteine; HCC: hepatocellular carcinoma; MASLD: Metabolic dysfunction-associated steatotic liver disease.

**Figure 2:**
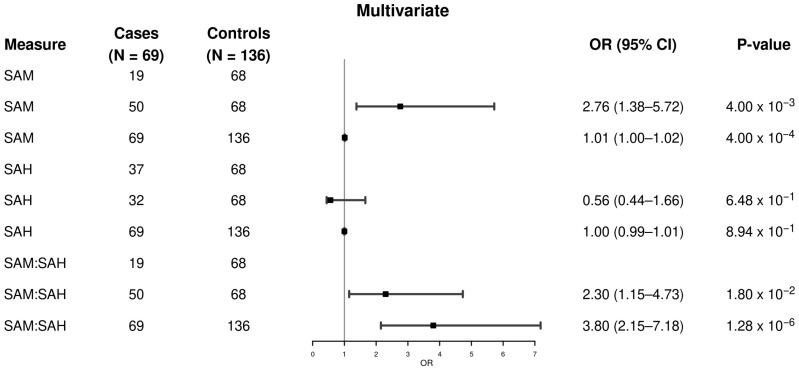
Multivariable-adjusted association of plasma SAM and SAH levels, and the SAM/SAH ratio with MASLD-HCC. The analysis included 205 samples (69 MASLD-HCC cases and 136 MASLD controls). Logistic regression models were used to calculate odds ratios, 95% confidence intervals, and p-values, adjusting for age (continuous), sex, body mass index (continuous), smoking status (never, former, current), and type 2 diabetes (yes, no). In the multivariable-adjusted models, elevated plasma SAM levels and SAM/SAH ratios were associated with higher odds of MASLD-HCC, while SAH levels were not associated with MASLD-HCC. CI: confidence interval; OR: odds ratio; SAM: S-adenosylmethionine; SAH: S-adenosylhomocysteine; HCC: hepatocellular carcinoma; MASLD: metabolic dysfunction-associated steatotic liver disease.

In Figure 1 and Figure 2, the study of methylation-related metabolites indicated substantial variations between MASLD-HCC patients and MASLD controls. Plasma SAM levels were considerably higher in cases than in controls, with a larger proportion of cases reporting SAM concentration above the median. In the univariable analysis (Figure 1), we found that having a higher SAM level is associated with higher odds of MASLD-HCC (OR = 2.63, 95% CI: 1.42–5.01, *p* = 0.002, ≥median vs. <median; OR continuous = 1.01, 95% CI: 1.00–1.02, *p* = 0.001). However, a higher SAH level was not associated with the odds of having MASLD-HCC (OR = 0.86, 95% CI: 0.48–1.54, *p* = 0.62, ≥median vs. <median; OR continuous = 1.00, 95% CI: 0.98–1.01, *p* = 0.47). By contrast, a higher SAM/SAH ratio was associated with higher odds of MASLD-HCC (OR = 2.63, 95% CI: 1.42–5.01, *p* = 0.002, ≥median vs. <median; OR continuous = 4.01, 95% CI: 2.47–6.88, *p* = 9.31 × 10^−8^).

In the multivariable model with adjustment for covariates such as age, sex, BMI, smoking history, and diabetes, (Figure 2), we found that higher SAM levels remained significantly associated with higher odds of MASLD-HCC (OR = 2.76, 95% CI: 1.38–5.72, *p* = 0.004, ≥median vs. <median; OR continuous = 1.01, 95% CI: 1.00–1.02, *p* = 0.004). We again did not find an association between a higher SAH level and MASLD-HCC (OR = 0.86, 95% CI: 0.44–1.66, *p* = 0.65, ≥median vs. <median; OR continuous = 1.00, 95% CI: 0.99–1.01, *p* = 0.89). By contrast, a higher SAM/SAH ratio was significantly associated with higher odds of MASLD-HCC (OR = 2.30, 95% CI: 1.15–4.73, *p* = 0.018, ≥median vs. <median; OR continuous = 3.80, 95% CI: 2.15–7.18, *p* = 1.28 × 10^−6^).

## 4. Discussion

In this pilot study, we investigated whether the risk of HCC in patients with MASLD was related to plasma levels of SAM, SAH, or the SAM/SAH ratio. Independent of known risk factors of HCC, such as age, sex, BMI, smoking status, and type 2 diabetes, we found that elevated plasma levels of SAM and the SAM/SAH ratio were each associated with higher odds of MASLD-HCC. However, SAH was not independently associated with the odds of MASLD-HCC. These results suggest that elevated SAM levels and the SAM/SAH ratio, which are precursor molecules of DNA methylation and are involved in several hepatic metabolic reactions, might play a role in HCC development in patients with MASLD. Collectively, this initial evidence indicates the SAM and SAM/SAH ratio could be potential markers of HCC development or progression and might be useful for enhancing risk models or early HCC detection models in patients with metabolic perturbations.

As the worldwide liver cancer epidemiology changes, HCC is increasingly occurring in the setting of MASLD. MASLD and alcohol-related liver diseases are now predicted to be the leading causes of liver cancer-related fatalities by 2040, especially in Western countries [29], since viral hepatitis is now very effectively managed by vaccinations for HBV and antiviral therapy for both HBV and HCV. This epidemiologic shift has fueled growing interest in understanding the metabolic and epigenetic mechanisms driving HCC development in patients with nonalcoholic, non-viral liver disease. Although SAM deficiency tends to favor the formation of liver cancer and its supplementation can inhibit hepatocarcinogenesis in experimental animals, there is limited evidence to support these conclusions in human populations [24]. However, current studies on humans have begun to clarify this relationship. The genome-wide DNA methylation patterns of blood leucocytes from MASLD-HCC patients and MASLD controls were assessed in multi-center research by Antwi et al. [4]. When paired with clinical information such as age, sex, diabetes status, and ethnicity, the study found a panel of 55 differentially methylated CpG sites that had a moderate predictive performance (AUC = 0.78) in differentiating HCC patients from controls [4]. These findings emphasize the need to understand the metabolic bases of those epigenetic alterations and the clinical significance of DNA methylation variations in MASLD-HCC.

Even though SAM is the universal methyl donor used for DNA methylation [7,8], methylation of DNA represents only a minor portion of hepatic SAM utilization [20,21]. In hepatocytes, SAM is extensively utilized in a wide range of transmethylation and metabolic reactions, including the biosynthesis of phosphatidylcholine through the PEMT pathway, as well as the generation of creatine, carnitine, glutathione, and polyamines [18,21,22,23]. Consequently, elevations in plasma SAM levels and SAM/SAH ratios observed among individuals with MASLD-HCC likely reflect broader dysregulation of hepatic one-carbon metabolism and methyl-group flux, rather than changes restricted to DNA methylation pathways only. From a biomarker perspective, the specific metabolic origin of SAM elevation may be less critical than the reproducibility and prognostic relevance of SAM and SAH measurements in the setting of metabolic perturbations. Within this context, our findings suggest that circulating SAM levels and the SAM/SAH ratio may serve as informative biomarkers of HCC, with potential utility for improving risk stratification and early detection strategies in patients with metabolic abnormalities.

Tang et al. investigated circulating one-carbon metabolites in patients with nonalcoholic fatty liver disease (NAFLD/MASLD) [30]. They found that as the severity of hepatic steatosis increased, serum SAH levels also increased, but the SAM/SAH ratio declined, implying a lower methylation capacity or a broader metabolic dysregulation in the liver [20,30,31]. These data indicate that a decreased SAM/SAH ratio may increase the risk of liver disease development. However, the study by Tang et al. was focused on NAFLD/MASLD as the endpoint, and not NAFLD/MASLD-related HCC or HCC in general, which is a major distinction from our study [30]. Furthermore, these results differ from what we observed in the present study, where greater SAM levels and a higher SAM/SAH ratio were both linked with higher odds of MASLD-HCC. This apparent discrepancy may be due to a change in disease stage (benign disease vs. malignancy), the extent of underlying metabolic dysregulation in patients across the studies, or the biological compartment examined (liver tissue vs. plasma). Our findings suggest that both plasma SAM and the SAM/SAH ratio are higher in MASLD-HCC patients, implying that SAM production may become dysregulated in the presence of hepatic tumors or during the transitional pre-malignant phase of MASLD to HCC. It is also plausible that SAM accumulation may occur due to poor methyl group utilization or methyltransferase inhibition rather than enhanced methylation activity. This is consistent with previous studies of metabolic rewiring in HCC cells, which include enhanced one-carbon metabolism and delayed methyl donor flow to allow rapid cell growth [32].

Notably, we found no statistically significant association between SAH levels and MASLD-HCC, contradicting previous theories that SAH is a cause of hepatic inflammation and liver damage. A recent prospective analysis of individuals with established HCC discovered that increased plasma SAH levels were associated with a worse outcome but not disease risk [14]. This lends credence to our hypothesis that SAH may be more relevant to HCC progression or severity than HCC onset, specifically in the metabolic disease context. The use of methylation markers as diagnostic or risk-prediction tools is gaining clinical attention. Our findings indicate that circulating SAM and the SAM/SAH ratio may also be useful indicators of MASLD patients’ progression to more advanced liver disease and could be integrated into multifactorial screening or disease stratification models.

Our study had several strengths and limitations. The strengths include evaluation of molecular disease markers in plasma samples in relation to HCC in a well-defined MASLD patient population, while eliminating the potential confounding effects of competing liver diseases (e.g., rare genetic diseases that predispose to HCC, HBV, HCV, and alcoholic liver disease) [3]. The multi-center, multidimensional approach incorporated data from locations spanning Europe and the United States, improving the generalizability of our findings. Furthermore, the one-to-two matched case–control design and adjustment for HCC risk factors add an additional layer of rigor to the study. The use of standardized mass spectrometry assays allowed consistent and repeatable measurement of methylation-related metabolites, which strengthened our biomarker analysis [14,33]. Moreover, by identifying high plasma SAM and SAM/SAH ratios as possible biomarkers for MASLD-related HCC, our study addressed a crucial unmet need for non-invasive risk stratification tools in this prevalent population. Limitations include the inability to make inferences on causality due to the observational study design, and it remains unclear whether elevated SAM and an increase in the SAM/SAH ratio contribute to HCC onset, or the presence of HCC alters the levels of these metabolites. Either scenario would have important implications for risk assessment and the refinement of early detection models, particularly if future large-scale studies confirm that alterations in SAM or the SAM/SAH ratio occur because of clinically inapparent HCC in patients with MASLD, potentially improving early detection of this frequently fatal cancer. Although the small sample size may restrict statistical power, generalizability was improved by utilizing an international multicenter cohort. It was impossible to rule out residual confounding from unmeasured patient characteristics, such as the influences of diet and medication use. Although all samples were stored at –80 °C until use, and the majority of study sites shipped whole blood to Mayo Clinic for plasma extraction and subsequent assays, two sites provided pre-extracted plasma samples. Quality control assessments did not reveal systematic differences in plasma metabolite levels across sites; however, potential variability introduced by differences in plasma processing protocols cannot be completely ruled out. Also, hepatic intracellular activity may be more accurately measured in liver tissue, although plasma tests offer easily accessible, minimally invasive indicators. Given the pilot nature of this study, we were unable to examine interactions between risk factors such as BMI, hypertension, and other cardiometabolic conditions. These relationships should be investigated in larger, follow-up studies with sufficient statistical power. Future prospective studies, including larger cohorts and integrated tissue analysis, would help clarify the causal role of SAM and SAH in HCC development.

## 5. Conclusions

In this study, we found that high plasma levels of SAM and the SAM/SAH ratio were associated with higher odds of MASLD-HCC, independent of known risk factors. These findings underscore the important role of precursors of DNA methylation and epigenetic dysregulation in MASLD-HCC development. SAM and the SAM/SAH ratio may serve as noninvasive biomarkers for HCC stratification models in patients with MASLD and might also enhance early detection models, addressing a critical gap in current prevention and surveillance strategies.

## Figures and Tables

**Table 1:** Characteristics of the study participants (N = 205).

	MASLD-HCC Cases (N = 69)	Metabolic Controls (N = 136)	*p*-Value ^b^
Age, years ^a^			0.402
Mean (SD)	68 (12)	67 (10)	
Sex			0.794
Female	21 (30%)	39 (29%)	
Male	48 (70%)	97 (71%)	
Site			5.78 × 10^−26^
Mayo Clinic	30 (44%)	125 (92%)	
Karolinska University Hospital	21 (30%)	3 (2%)	
BCLC, Barcelona, and IISB, San Sebastian, Spain	18 (26%)	8 (6%)	
BMI, kg/m^2^			
≤24.9	9 (13%)	17 (13%)	0.000001
25–29.9	42 (61%)	35 (26%)	
≥30	18 (26%)	84 (62%)	
Mean (SD)	29 (4)	32 (7)	0.0000781
Diabetes Mellitus, Yes	47 (68%)	75 (55%)	0.074
Smoking			0.036
Never	24 (35%)	66 (50%)	
Former	38 (55%)	63 (47%)	
Current	7 (10%)	4 (3.0%)	
Unknown	0	3	
SAM, nmol/L			0.001
Mean (SD)	121 (60)	96 (43)	
Median (Q1, Q3)	103 (86, 132)	87 (67, 118)	
SAH, nmol/L			0.113
Mean (SD)	63 (30)	65 (22)	
Median (Q1, Q3)	59 (45, 71)	61 (51, 73)	
SAM/SAH, nmol/L			6.42 × 10^−7^
Mean (SD)	2.09 (0.88)	1.48 (0.47)	
Median (Q1, Q3)	1.98 (1.33, 2.68)	1.44 (1.21, 1.69)	

^a^ Chronological age at diagnosis for cases and at recruitment for controls. ^b^ *p*-values were calculated using Wilcoxon’s test for continuous variables (age and BMI) and chi-square test for categorical variables. Abbreviations: BCLC, Barcelona Clinic Liver Cancer Group, Barcelona, Spain; BMI, body mass index; Spain; IISB, Instituto de Investigación Sanitaria Biodonostia Research Institute, Donostia University Hospital, San Sebastian, Spain.

## Data Availability

The data can be made available to interested researchers upon reasonable request to the corresponding author.

## Abbreviations

The following abbreviations are used in this manuscript:

AUC	Area Under the Curve
BCLC	Barcelona Clinic Liver Cancer Group
BMI	Body mass index
CI	Confidence interval
CpG	Cytosine-phosphate-Guanine
DNA	Deoxyribonucleic acid
ER	Endoplasmic reticulum
HBV	Hepatitis B virus
HCC	Hepatocellular carcinoma
HCV	Hepatitis C
IBIS	Institute of Biomedicine of Sevilla
IISB	Instituto de Investigación Sanitaria Biogipuzkoa
IRB	Institutional Review Board
MASLD	Metabolic dysfunction-associated steatotic liver disease
